# MKL1-induced lncRNA SNHG18 drives the growth and metastasis of non-small cell lung cancer via the miR-211-5p/BRD4 axis

**DOI:** 10.1038/s41419-021-03399-z

**Published:** 2021-01-26

**Authors:** Huijie Fan, Jing Yuan, Yaqing Li, Yongxu Jia, Jing Li, Xiaofeng Wang, Xingya Li

**Affiliations:** Department of Oncology, The First Affiliated Hospital of Zhengzhou University, Zhengzhou, China

**Keywords:** Non-small-cell lung cancer, Lung cancer

## Abstract

Megakaryocytic leukemia 1 (MKL1) is a key transcription factor involved in non-small cell lung cancer (NSCLC) growth and metastasis. Yet, its downstream target genes, especially long non-coding RNA (lncRNA) targets, are poorly investigated. In this study, we employed lncRNA array technology to identify differentially expressed lncRNAs in NSCLC cells with or without overexpression of MKL1. Candidate lncRNAs were further explored for their clinical significance and function in NSCLC. The results showed that MKL1 promoted the expression of lncRNA SNHG18 in NSCLC cells. SNHG18 upregulation in NSCLC specimens correlated with lymph node metastasis and reduced overall survival of NSCLC patients. SNHG18 expression served as an independent prognostic factor for NSCLC. Knockdown of SNHG18 blocked MKL1-induced growth and invasion of NSCLC cells in vitro. Animal studies validated the requirement for SNHG18 in NSCLC growth and metastasis. Moreover, overexpression of SNHG18 promoted NSCLC cell proliferation and invasion. Mechanically, SNHG18 exerted its prometastatic effects on NSCLC cells through repression of miR-211-5p and induction of BRD4. Clinical evidence indicated that SNHG18 expression was negatively correlated with miR-211-5p expression in NSCLC tissues. Altogether, SNHG18 acts as a lncRNA mediator of MKL1 in NSCLC. SNHG18 facilitates NSCLC growth and metastasis by modulating the miR-211-5p/BRD4 axis. Therefore, SNHG18 may be a potential therapeutic target for the treatment of NSCLC.

## Introduction

Megakaryocytic leukemia 1 (MKL1), also known as myocardin-related transcription factor A (MRTF-A), is a transcription factor that is ubiquitously expressed in various cell types^1,2^. MKL1 has been shown to play an important role in multiple biological processes, such as cardiac development, inflammation, diabetic nephropathy, lung fibrosis, and tumor development^3–7^. When MKL1 is translocated to the nucleus, it can interact with other transcription factors and then initiate the transcription of downstream target genes. For instance, MKL1 forms a complex with serum response factor (SRF) to promote the transcription of alpha-smooth muscle actin in response to transforming growth factor-beta1 (TGF-β1) treatment^8^. Similarly, MKL1 forms a complex with NF-κB/p65 to drive the transcription of adhesion molecules in vascular endothelial cells and pro-inflammatory genes in macrophages^9,10^. MKL1 can also induce the transactivation of a lot of cancer-related genes such as MMP2 and GLI1^7,11^. These studies indicate that many protein-coding genes can be transactivated by MKL1.

Long non-coding RNAs (lncRNAs) are a large class of non-coding RNAs of longer than 200 nucleotides^12,13^. Dysregulation of lncRNAs is detected in a wide range of cancer types. For instance, lncRNA MEG3 is downregulated in esophageal cancer and gallbladder cancer^14,15^. Emerging evidence has indicated the pivotal role of lncRNA small nucleolar RNA host genes (SNHGs) in cancer progression^16–19^. SNHG5 promotes breast cancer proliferation and cell cycle progression^16^. Overexpression of SNHG6 enhances the proliferation and invasion of colorectal cancer cells^17^. SNHG18 is upregulated and induces radioresistance in glioma^18^. LncRNAs can interact with microRNAs (miRNAs) and interfere with the regulation of target genes by miRNAs^16,17^. For instance, lncRNA SNHG6 upregulates EZH2 by sponging miR-26a/b and miR-214 in colorectal cancer cells^17^. Therefore, identification of key lncRNAs involved in cancer progression is of significance.

Non-small cell lung cancer (NSCLC) accounts for over 80% of all lung cancer cases^20^. Although multiple novel therapies have been developed to treat NSCLC, the 5-year survival rate of patients with advanced NSCLC is still low^21,22^. Previously, it has been reported that MKL1 enhances NSCLC cell invasion and metastasis by activating MMP9 transcription^23^. However, MKL1-mediated regulation of non-coding genes is relatively less investigated. In this study, we focused on the downstream lncRNA(s) mediating the oncogenic role of MKL1 in NSCLC growth, invasion, and metastasis.

## Materials and methods

### Cell culture

Human NSCLC cell lines (A549, H1299, H23, H460, and H1792) were maintained in Dulbecco’s modified Eagle’s medium supplemented with 10% fetal bovine serum (FBS; Life Technologies, Grand Island, NY, USA). BEAS-2B human bronchial epithelial cells were grown in BEBM basal medium (Lonza, Walkersville, MD, USA) with 10% FBS in a humidified incubator at 37 °C with 5% CO_2_. These cell lines were validated using the short tandem repeat profiling technique.

### Patients and tissue specimens

We collected 63 paired NSCLC and adjacent noncancerous lung tissues from NSCLC patients undergoing tumor resection. None of them received any anticancer therapy before surgery.

### Quantitative PCR analysis and lncRNA PCR array assay

Total RNA of tissues or cells was isolated using Trizol reagent (Takara, Dalian, China). RNA was reversely transcribed into cDNA using the QuantiTect Reverse Transcription Kit from Qiagen (Hilden, German). Quantitative PCR analysis was performed using the SYBR Premix Ex Taq II (Takara). The primer sequences are shown in Supplementary Table S1. Glyceraldehyde 3-phosphate dehydrogenase (GAPDH) was used as a normalization control. Quantification of miRNAs was conducted using the miRCURY LNA miRNA PCR Assay kit (Qiagen), according to the manufacturer’s instructions. The relative expression of miRNAs was normalized against U6 expression.

To identify the lncRNAs that mediate the tumor-promoting activity of MKL1, we performed lncRNA PCR array assays in MKL1-overexpressing NSCLC cells. The lncRNA PCR array included 84 key lncRNAs involved in cancer cell proliferation, survival, migration, and invasion. Quantitative PCR was conducted as described above. Expression of each lncRNA was normalized to the average value of 5 housekeeping genes. Results are expressed as fold change relative to the vector group.

### Plasmids and oligonucleotides

The cDNA sequences expressing MKL1, SNHG18, and BRD4 were cloned into the pcDNA3.1 (+) vector. Short hairpin RNAs (shRNAs) targeting *MKL1* and *SNHG18* were synthesized by Sangon Biotechnology (Shanghai, China) and inserted into the pLKO.1 vector. BRD4 small interfering RNAs (siRNAs) and negative control siRNA were synthesized by Sangon Biotechnology. The target sequences of these shRNAs and siRNAs are shown in Supplementary Table S2. miR-211-5p mimic was purchased from Sigma-Aldrich (St. Louis, MO, USA) and anti-miR-211-5p inhibitor from Thermo Fisher Scientific (Rockford, IL, USA) (Supplementary Table S3). The constructs were transfected with Lipofectamine 3000 transfection reagent (Thermo Fisher Scientific). SNHG18-depleted A549 and H1299 cell lines were generated by selecting in the presence of 2 μg/mL puromycin (Thermo Fisher Scientific) for 3-5 days.

### Cell proliferation assay

NSCLC cells transfected with indicated constructs (1 × 10^4^) were cultured for 3 days. Cell counting using a hemocytometer was done every day. The proliferation curves were plotted based on cell number.

### Colony formation assay

Cells were plated at 600 wells/well and cultured for 10–14 days. The cells were fixed and stained with 0.1% crystal violet. Colonies were photographed and manually counted.

### Wound-healing assay

Cells were plated onto 12-well plates (5 × 10^5^ cells/well) and grew to confluence. The cell monolayer was scratched using a sterile pipette tip. Cell debris was removed, and the attached cells were cultured for 24 h. The percentage of wound closure was evaluated from 5 random microscopic fields.

### Transwell invasion assay

Transwell chambers (8 μm in pore size; Corning, Tewksbury, MA, USA) were coated with Matrigel (BD Biosciences, Franklin Lakes, NJ, USA). Cells (5 × 10^4^) in serum-free medium were added in the upper chamber, and the lower chamber was filled with culture medium with 10% FBS. After incubation for 24 h, the cells that invaded through the Matrigel membrane were stained with 0.1% crystal violet and counted under an inverted microscope.

### Animal studies

Male BALB/c nude mice (4 week old) were acclimated to the laboratory environment for 1 week. Stably transfected NSCLC cell lines (2 × 10^6^) were injected subcutaneously into the nude mice (*n* = 5 for each group). Tumor volume was recorded every week. Four weeks later, the mice were sacrificed. The tumors were excised and weighed. Immunostaining for ki-67 was performed on tumor sections using an anti-ki-67 antibody (1:200 dilution; Abcam, Cambridge, UK). Immunostaining results were scored by two experienced pathologists in a blind manner. Staining intensity was scored as 0 (no staining), 1 (mild staining), 2 (moderate staining), and 3 (strong staining). Staining proportion was scored as 0 (<5% of stained cells), 1 (5–25%), 2 (26–50%), 3 (51–75%) and 4 (>75%). The final ki-67 staining score was calculated by multiplying the staining score and the proportion score.

To generate a lung metastasis model, luciferase-labeled stable A549 and H1299 cells (5 × 10^6^) were injected into the tail veins of nude mice (*n* = 5 for each group). Six weeks later, D-luciferin was intraperitoneally injected, and bioluminescence imaging was done. Lung tissues were removed and processed for histological analysis with haematoxylin and eosin (H&E) staining.

### Chromatin immunoprecipitation (ChIP) assay

ChIP assay was conducted as described previously^24^. Briefly, cells were fixed with 1% formaldehyde (Sigma-Aldrich) and lysed in ChIP lysis buffer (Santa Cruz Biotechnology, Santa Cruz, CA, USA). DNA was sheared by sonication to the length of ~500 bp. The fragmented DNA sample was incubated with anti-MKL1 antibody (Cell Signaling Technology, Danvers, MA, USA) or control IgG. The immunocomplexes were captured by protein G beads. DNA was extracted using the QIAquick PCR purification kit (Qiagen) and analyzed by quantitative PCR. The primers are listed in Supplementary Table S1.

### Western blot analysis

Cells were lysed with RIPA buffer (Sigma-Aldrich) containing protease inhibitor cocktail (Thermo Fisher Scientific). Protein concentration was determined by a commercial BCA assay kit (Sigma-Aldrich). The cell extracts were separated by sodium dodecyl sulfate-polyacrylamide gel electrophoresis. The following primary antibodies were used: anti-BRD4 (1:1000 dilution; Thermo Fisher Scientific) and anti-GAPDH (1:5000 dilution; Abcam). Horseradish peroxidase-conjugated IgG was used as secondary antibody. The blots were developed using the Pierce ECL Western Blotting Substrate (Thermo Fisher Scientific). Densitometric analysis of protein bands was then performed.

### Luciferase reporter assay

To prepare promoter reporter constructs, different fragments of the *SNHG18* promoter (−1900 to +100 bp) were cloned to the upstream of firefly luciferase of pGL3 vector. *BRD4* 3′-untranslated region (3′-UTR) reporter was generated by cloning the 3′-UTR of *BRD4* to the downstream of the luciferase gene. Luciferase reporter assay was performed as described previously^25^. Briefly, cells were co-transfected with the luciferase reporter vectors, *Renilla* luciferase expression vector (as a transfection control), and indicated expression constructs. Twenty-four hours later, cells were lysed. Relative luciferase activities were determined using the Dual-luciferase Reporter Assay System (Promega, Madison, WI, USA).

### RNA immunoprecipitation (RIP) assay

RIP assay was conducted as described previously^26^. In brief, H23 cells were lysed in RIP lysis buffer from the EZ-Magna RIP Kit (Millipore, Billerica, MA, USA). The cell lysates were incubated with anti-Ago2 antibody or control IgG (Thermo Fisher Scientific) conjugated to magnetic beads. The immunoprecipitated RNA was purified and analyzed by quantitative PCR.

### Statistical analysis

Data are expressed as mean ± standard deviation (SD). Normal distribution of data was confirmed by the Shapiro–Wilk test. Student’s *t*-test was performed to compare the statistical significance of the differences between two groups. Differences among multiple groups were examined by one-way analysis of variance (ANOVA) followed by Bonferroni’s test. At least three independent biological repeats were used for in vitro experiments. Gene expression correlation was evaluated using Pearson’s correlation analysis. Survival analysis was performed using a Kaplan–Meier plot and Log-rank test. Multivariate Cox regression analysis was used to evaluate the prognostic significance of SNHG18. *P*-values < 0.05 were considered significant.

## Results

### SNHG18 is transactivated by MKL1 in NSCLC cells

**Fig. 1:**
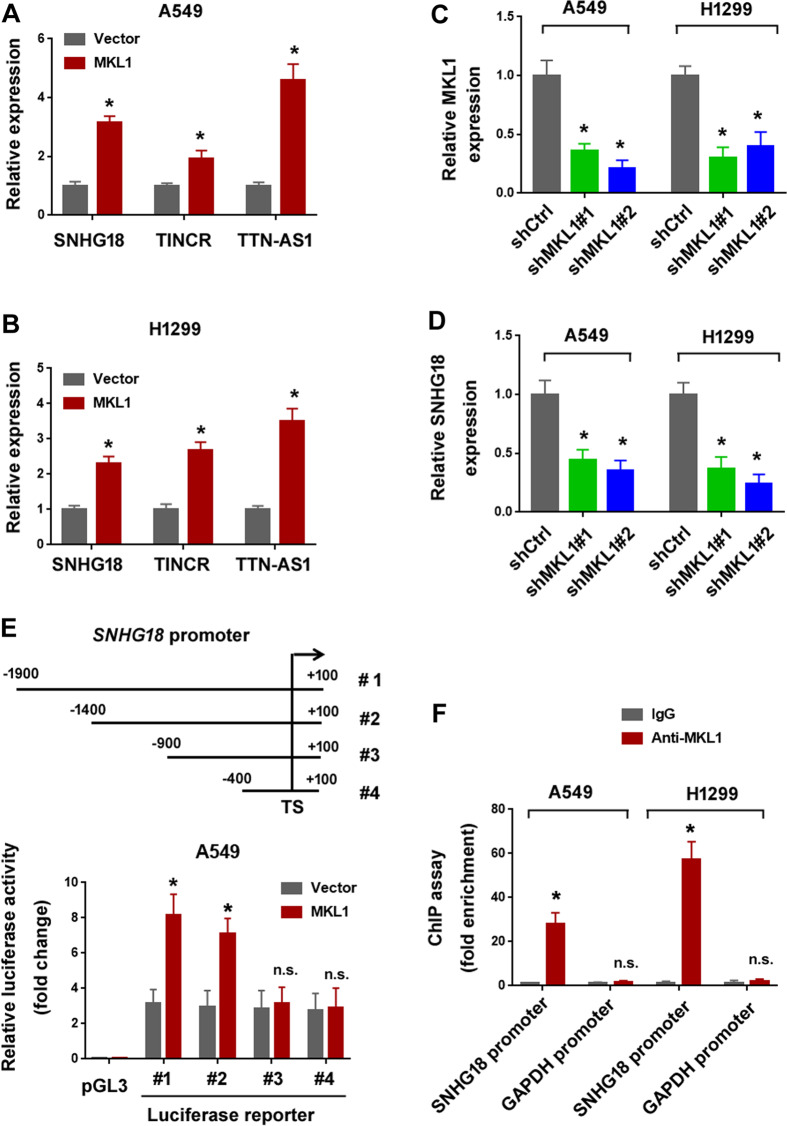
SNHG18 is transactivated by MKL1 in NSCLC cells. **A**, **B** Quantitative PCR analysis of indicated lncRNAs in **A** A549 and **B** H1299 cells transfected with empty vector or MKL1-expressing vector. *P < 0.05 *vs*. vector, Student’s *t*-test. *n* = 3. **C** Quantitative PCR analysis of MKL1 mRNA in NSCLC cells transfected with negative control shRNA (shCtrl) or 2 different shRNAs targeting MKL1 (shMKL1#1 and shMKL1#2). *P < 0.05 vs. shCtr, one-way ANOVA followed by Bonferroni’s test. *n* = 3. **D** Quantitative PCR analysis of SNHG18 expression in MKL1 shRNA-transfected NSCLC cells. *P < 0.05 vs. shCtr, one-way ANOVA followed by Bonferroni’s test. *n* = 3. **E** A549 cells were transfected with SNHG18 promoter reporters together with MKL1-expressing vector, and luciferase activities were measured 24 h after transfection. *P < 0.05 vs. vector, Student’s *t*^-^test. n.s. indicates no significance rela*t*ive to vector. *n* = 3. **F** ChIP assay showed the occupancy of MKL1 protein on the promoter of *SNHG18* (−1269 to −1072 bp) but not *GAPDH* (−781 to −435 bp). *P < 0.05 vs. control IgG, Student’s *t*-test. n.s. indicates no significance relative to control IgG. *n* = 3.

To identify MKL1-induced lncRNAs in NSCLC, we profiled 84 cancer-related lncRNAs using functional lncRNA arrays in both A549 and H1299 cells transfected with empty vector or MKL1-expresssing plasmid. We found that 3 lncRNAs were consistently upregulated in MKL1-overexpressing A549 and H1299 cells relative to control cells (Fig. 1A, B). Although 5 lncRNAs were downregulated in MKL1-overexpressing cells (3 in A549 cells and 2 in H1299 cells), none of them was reduced in both A549 and H1299 cells (data not shown). To confirm the upregulation of the 3 lncRNAs by MKL1, we performed MKL1 knockdown experiments in H1299 cells, which had high levels of endogenous MKL1. Transfection with 2 individual shRNAs led to efficient knockdown of MKL1 (Fig. 1C). Depletion of MKL1 caused a significant decrease in the level of SNHG18, but not the other 2 lncRNAs (Fig. 1D and Supplementary Fig. 1). To validate MKL1-mediated transactivation of SNHG18, we generated a series of truncated *SNHG18* promoter-luciferase constructs. Luciferase reporter assay demonstrated that the region (−1400 to −900 bp) of *SNHG18* promoter contained MKL1 responsive site (Fig. 1E). ChIP assay further revealed the enrichment of MKL1 protein on *SNHG18* promoter (Fig. 1F).

### SNHG18 is upregulated and predicts poor prognosis in NSCLC

**Fig. 2:**
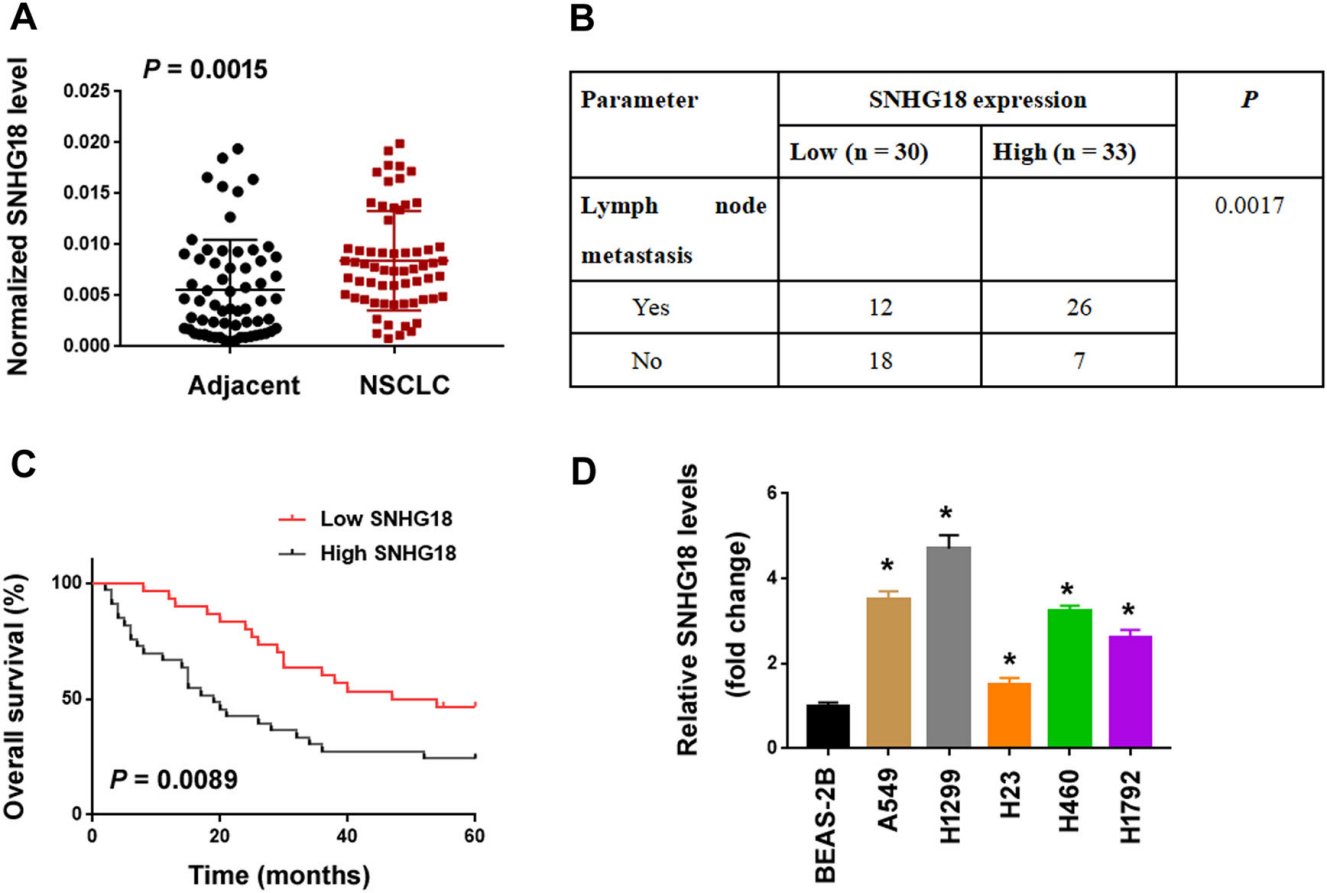
SNHG18 is upregulated and predicts poor prognosis in NSCLC. **A** Relative SNHG18 expression measured in 63 paired NSCLC and adjacent noncancerous tissues. *P* value, Student’s *t*-test. **B** Analysis of the relationship between SNHG18 expression and lymph node metastasis. **C** Kaplan–Meier survival curves were performed to determine the impact of SNHG18 expression on the overall survival of NSCLC patients. *P* value, log-rank test. **D** SNHG18 levels in NSCLC cell lines and BEAS-2B lung epithelial cells analyzed by quantitative PCR. ^*^P < 0.05 vs. BEAS-2B, one-way ANOVA followed by Bonferroni’s test.

To determine the expression and clinical significance of SNHG18 in NSCLC, we collected 63 pairs of NSCLC and adjacent noncancerous lung tissues and measured the expression of SNHG18 by real-time PCR analysis. The results showed that SNHG18 expression levels were significantly greater in NSCLC tissues than those in adjacent normal tissues (*P* = 0.0015; Fig. 2A). Moreover, high SNHG18 expression was significantly correlated with lymph node metastasis (*P* = 0.0017; Fig. 2B). Kaplan–Meier analysis showed that patients with high tumoral SNHG18 expression had significantly shorter overall survival than patients with low SNHG18 expression (*P* = 0.0089; Fig. 2C). Multivariate Cox regression analysis demonstrated that SNHG18 expression (*P* = 0.004) and lymph node metastasis (*P* = 0.029) were independent risk factors for NSCLC patients (Table 1).

**Table 1:** Multivariate Cox regression analysis for the 63 patients with NSCLC.

Variable	Hazard ratio	95% CI	*P*
LN metastasis (yes vs. no)	2.004	1.129-3.327	**0.029**
TNM stage (III vs. I–II)	1.216	0.815–2.836	0.084
SNHG18 expression (high vs. low)	2.459	1.584–4.971	**0.004**

*CI* confidence interval, *LN* lymph node.

*P* values in bold indicates statistical significance.

### Ablation of SNHG18 restrains MKL1-induced growth and invasion of NSCLC cells

**Fig. 3:**
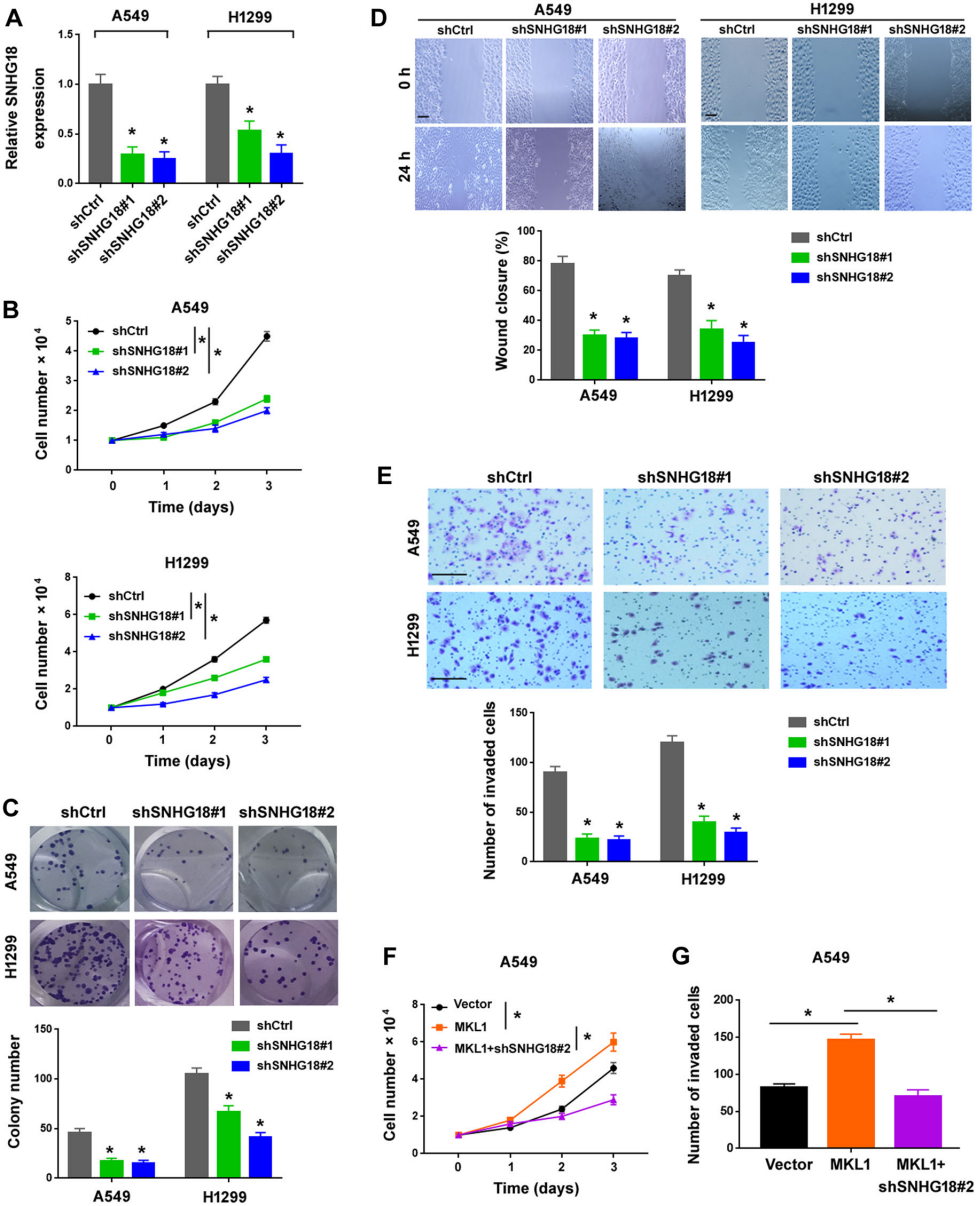
SNHG18 knockdown suppresses MKL1-induced growth and invasion of NSCLC cells. **A** Quantitative PCR analysis of SNHG18 expression in NSCLC cells transfected with negative control shRNA (shCtrl) or two different shRNAs targeting SNHG18 (shSNHG18#1 and shSNHG18#2). *n* = 3. **B** The proliferation of NSCLC cells transfected with indicated shRNAs was analyzed by direct cell counting. *n* = 4. **C** SNHG18 knockdown reduced the capability of clone formation by NSCLC cells. *n* = 3. **D** SNHG18 knockdown inhibited the migration of NSCLC cells, as determined by wound-healing assay. Bottom, bar graphs showing the quantitative results from three independent experiments. Scale bar = 300 μm. *n* = 3. **E** Reduction of cell invasion in NSCLC cells transfected with indicated shRNAs. Scale bar = 100 μm. *P < 0.05 vs. shCtrl. *n* = 3. **F** K*n*ockdown of SNHG18 blocked MKL1-induced cell proliferation in A549 cells. *n* = 4. **G** Assessment of cell invasion in A549 cells transfected with indicated constructs. *P < 0.05. In all panels, one-way ANOVA and Bonferroni’s tests were performed. *n* = 3.

To clarify the role of SNHG18 in NSCLC, we used shRNA technology to knockdown the expression of SNHG18 in both A549 and H1299 cells. The 2 NSCLC cell lines displayed relatively higher levels of SNHG18 than BEAS-2B bronchial epithelial cells (Fig. 2D). The knockdown efficiency of SNHG18 was validated by real-time PCR analysis (Fig. 3A). Notably, depletion of SNHG18 significantly inhibited NSCLC cell proliferation, as determined by direct cell counting (Fig. 3B). Colony formation assay demonstrated that depletion of SNHG18 significantly suppressed the colony-forming ability of NSCLC cells (Fig. 3C). Wound-healing assay showed that SNHG18-depleted A549 and H1299 cells had a significantly reduced migratory capacity than control cells (Fig. 3D). Transwell invasion assay demonstrated that knockdown of SNHG18 led to a decrease in the invasive potential of A549 and H1299 cells (Fig. 3E). We also explored the impact of SNHG18 silencing on MKL1-mediated aggressiveness of NSCLC cells. Interestingly, knockdown of SNHG18 abrogated MKL1-induced proliferation (Fig. 3F) and invasion (Fig. 3G) of NSCLC cells.

### Depletion of SNHG18 blocks NSCLC tumorigenesis and metastasis in vivo

**Fig. 4:**
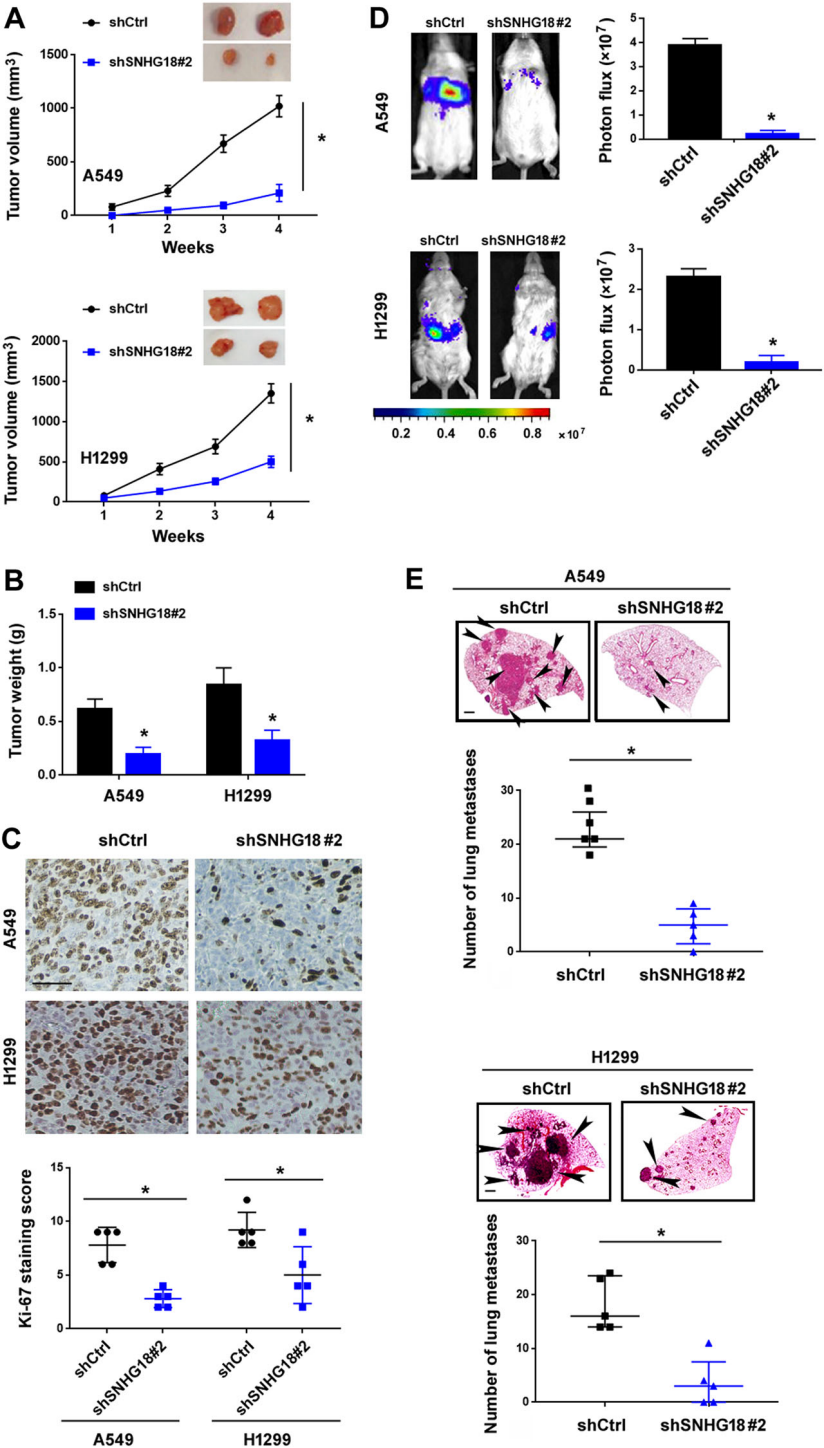
SNHG18 depletion restrains tumorigenesis and metastasis of NSCLC cells in vivo. **A** NSCLC cells with SNHG18 depletion were subcutaneously implanted to nude mice, and tumor growth was examined. Top, representative subcutaneous xenograft tumors from 2 mice of each group are shown. *n* = 5. **B** Tumor weight was measured 4 weeks after NSCLC cell implantation. *n* = 5. **C** Ki-67 immunostaining in tumor sections. *Top*, representative images of ki-67 staining. Scale bar = 100 μm. Bottom panels show ki-67 staining scores. *n* = 5. **D** In vivo bioluminescence imaging showed that knockdown of SNHG18 reduced the metastatic capacity of A549 and H1299 cells. Left, representative bioluminescent imaging of mice injected with luciferase-labeled A549 and H1299 cells. *n* = 5. **E** H&E-stained sections of the lung tissue collected from nude mice injected with SNHG1-depleted A549 and H1299 cells. Arrowheads indicate metastatic lesions in the lung. Bottom, quantification of metastatic lesions from 5 mice is shown. Scale bar = 100 μm. *P < 0.05 vs. shCtrl. In all panels, Student’s *t*-tests were performed. *n* = 5.

In vivo studies further revealed that the subcutaneous xenograft tumors formed by SNHG18-depleted cells had a slower growth rate than those by control shRNA-transfected cells (Fig. 4A). Tumor weight was lower in the SNHG18 knockdown group than in the control group (Fig. 4B). Ki-67 staining score was lower in the SNHG18 knockdown group than that in the control group (Fig. 4C). Next, we established a mouse lung metastasis model to validate whether depletion of SNHG18 can restrain the formation of metastatic lesions. In vivo bioluminescence imaging revealed that mice injected with A549-Luc-shSNHG18 cells had significantly reduced metastasis in the lung, compared to those injected with control A549-Luc cells (Fig. 4D). Similarly, SNHG18 knockdown attenuated metastatic potential of H1299 cells in vivo (Fig. 4D). Histological analysis confirmed a marked decline in the number of lung nodules in the shSNHG18 group (Fig. 4E).

### Overexpression of SNHG18 enhances the malignant behaviors of NSCLC cells

**Fig. 5:**
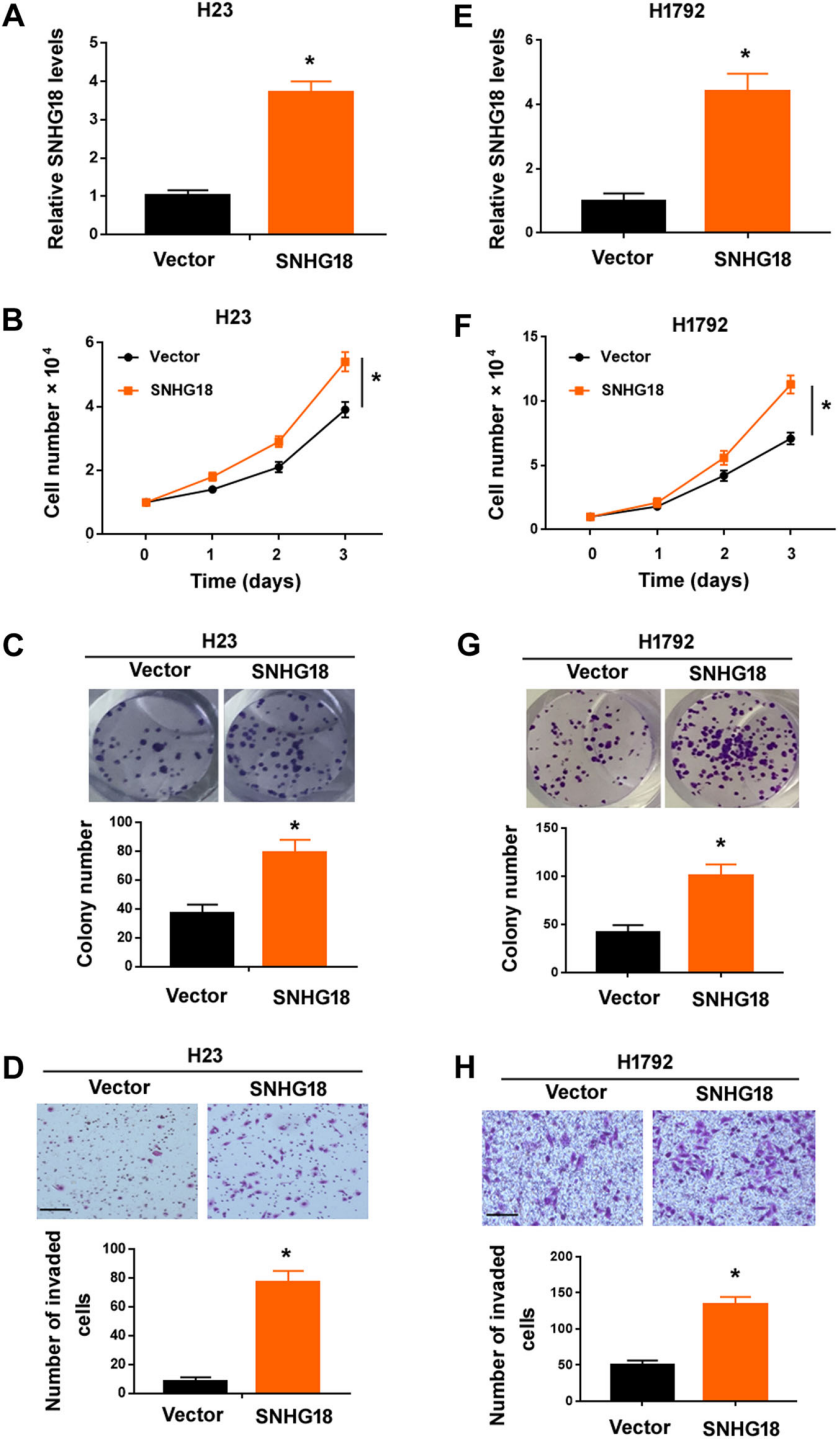
Overexpression of SNHG18 promotes NSCLC cell proliferation and invasion in vitro. **A** Quantitative PCR analysis of SNHG18 in H23 cells transfected with empty vector or SNHG18-expressing vector. *n* = 3. **B** Ectopic expression of SNHG18 increased the proliferation of H23 cells. *n* = 4. **C** Colony formation assay was performed to determine the ability of SNHG18-overexpressing H23 cells in forming colonies. *n* = 3. **D** Transwell invasion assay was used to assess the invasive ability of H23 cells with or without overexpression of SNHG18. *n* = 3. **E** Analysis of SNHG18 expression in H1792 cells. *n* = 3. **F**–**H** Overexpression of SNHG18 enhanced the **F** proliferation (*n* = 4), **G** colony formation (*n* = 3), and **H** invasion (*n* = 3) of H1792 cells. Scale bar = 100 μm in (**D**, **H**). *P < 0.05 *vs*. vector. In all panels, Student’s *t*-tests were performed.

To validate the role of SNHG18 in NSCLC, we performed gain-of-function experiments in both H23 and H1792 cells, which had relatively low levels of endogenous SNHG18. Enforced expression of SNHG18 (Fig. 5A) stimulated the proliferation (Fig. 5B) and colony formation (Fig. 5C) in H23 cells. Moreover, the invasive ability of H23 cells were increased when SNHG18 was overexpressed (Fig. 5D). Similarly, SNHG18 overexpression also augmented the proliferation, colony formation, and invasion of H1792 cells (Fig. 5E–H).

### SNHG18 interacts with miR-211-5p and antagonizes its inhibitory effects against NSCLC

**Fig. 6:**
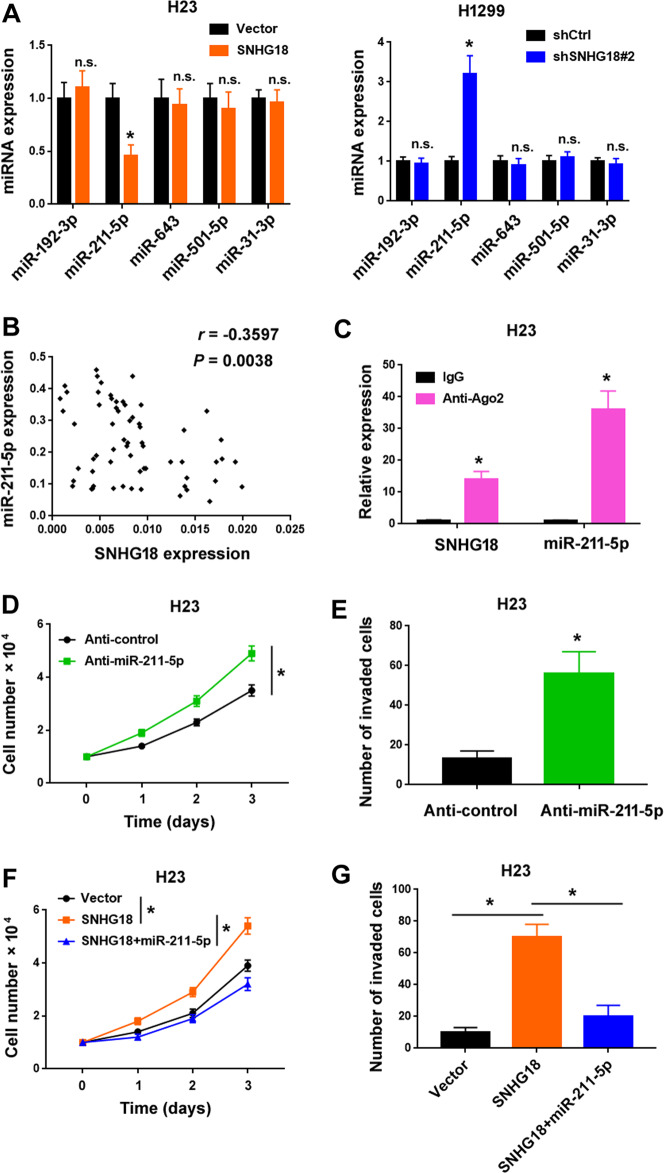
SNHG18 interacts with miR-211-5p and antagonizes its inhibitory effects against NSCLC. **A** Quantitative PCR analysis of indicated miRNAs in H23 and H1299 cells transfected with indicated constructs. n.s. indicates no significance. *P < 0.05 vs. vector or shCtrl. *n* = 3. **B** A*n*alysis of the correlation between SNHG18 and miR-211-5p expression in 63 NSCLC cases. **C** Ago2 RIP assay showed that SNHG18 and miR-211-5p were enriched in Ago2 immunoprecipitates from H1299 cells. *n* = 3. **D**, **F** Assessment of the proliferation of H23 cells transfected with indicated constructs. *P < 0.05 by one-way ANOVA and Bonferroni’s test in (**F**). *n* = 4. **E**, **G** Measurement of the invasion of H23 cells transfected with indicated constructs. *P < 0.05 by one-way ANOVA and Bonferroni’s test in (**G**). *n* = 3. In all panels in this figure, Student’s *t*-tests were performed unless otherwise described.

To uncover the mechanism for the oncogenic activity of SNHG18, we performed an in-silico screening for potential miR targets of SNHG18. Five candidates with high predicted scores, i.e., miR-192-3p, miR-211-5p, miR-643, miR-501-5p, and miR-31-3p, were selected for further validation. We found that overexpression of SNHG18 decreased the expression of miR-211-5p in H23 cells, whereas knockdown of SNHG18 resulted in an increase of miR-211-5p expression in H1299 cells (Fig. 6A). In contrast, the levels of miR-192-3p, miR-643, miR-501-5p, and miR-31-3p remained unchanged when SNHG18 was overexpressed or knocked down (Fig. 6A). Clinically, there was an inverse correlation between SNHG18 and miR-211-5p in NSCLC specimens (Fig. 6B). We then performed RNA pull-down experiments with antibodies against Ago2, a key component of the RNA-induced silencing complex (RISC)^27^. As a consequence, SNHG18 and miR-211-5p were enriched in Ago2 immunoprecipitates from H1299 cells (Fig. 6C), suggesting that SNHG18 and miR-211-5p are in the same RISC.

Next, we checked whether SNHG18-mediated aggressive phenotype is attributable to downregulation of miR-211-5p. Similar to the findings in SNHG18-overexpressing NSCLC cells, knockdown of miR-211-5p with specific anti-miR-211-5p inhibitors remarkably accelerated the proliferation (Fig. 6D) and invasion (Fig. 6E) of NSCLC cells. Co-expression of miR-211-5p reversed the oncogenic effect of SNHG18 on NSCLC cells (Fig. 6F, G).

### BRD4 serves as a functional target of miR-211-5p

**Fig. 7:**
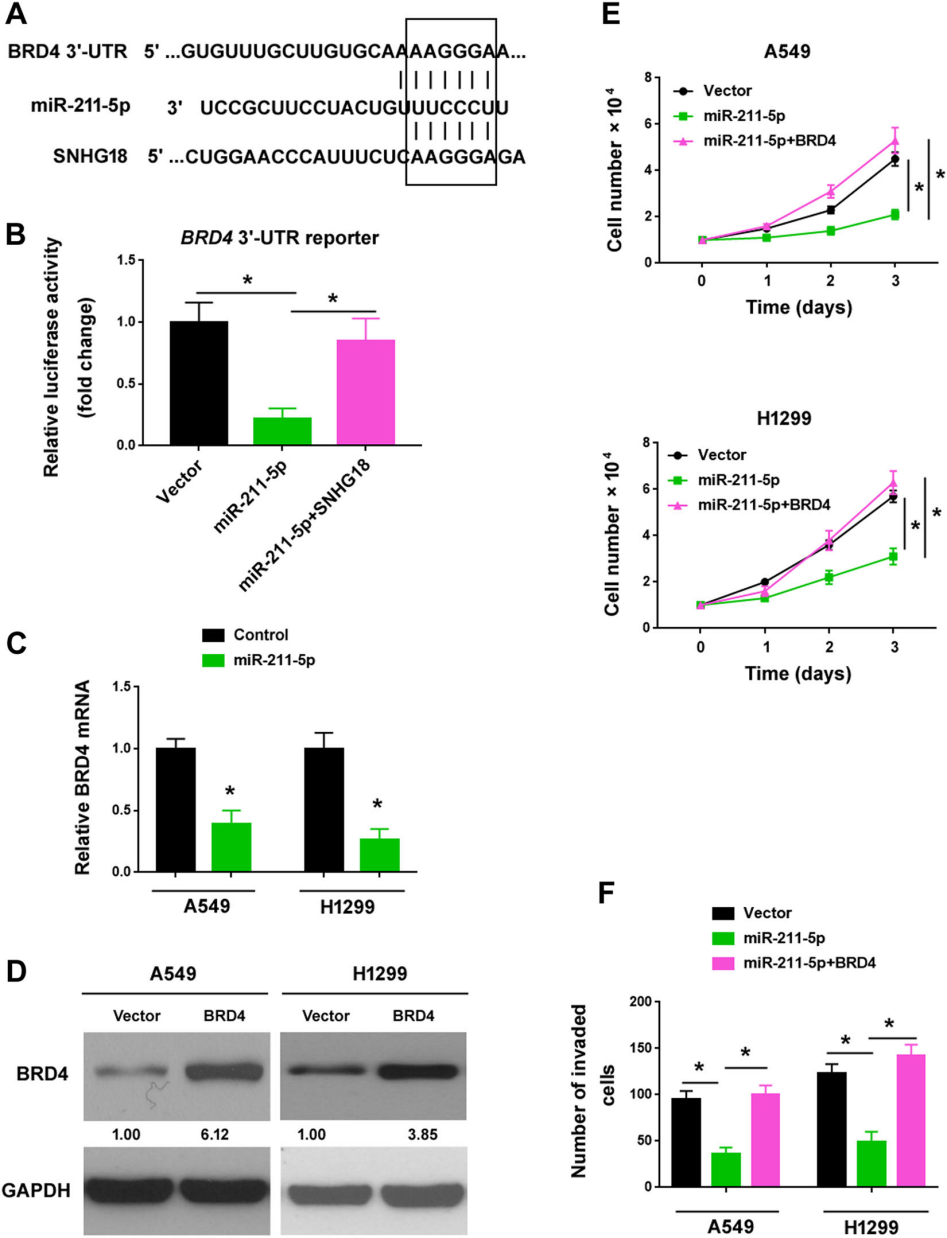
BRD4 serves as a functional target of miR-211-5p. **A** Bioinformatic analysis indicated the potential target sites for miR-211-5p in BRD4 and SNHG18. **B** Luciferase reporter assay showed that miR-211-5p repressed the expression of the *BRD4* 3’-UTR reporter. Overexpression of SNHG18 rescued miR-211-5p-mediated repression of *BRD4* 3’-UTR reporter. *n* = 3. **C** Quantitative PCR analysis of BRD4 mRNA in A549 and H1299 cells transfected with control mimic or miR-211-5p mimic. *P < 0.05 vs. control mimic, Student’s *t*-test. *n* = 3. **D** Western blot analysis of BRD4 protein expression in NSCLC cells transfected with empty vector or BRD4-expressing vector. **E**, **F** Assessment of **E** cell proliferation and **F** invasion in NSCLC cells transfected with indicated constructs. *P < 0.05. *n* = 3. In all panels in this figure, one-way ANOVA and Bonferroni’s tests were performed unless otherwise described.

Next, we tested the hypothesis that SNHG18 acts as a sponge of miR-211-5p to derepress the expression of miR-211-5p target genes. Bioinformatic analysis revealed that many genes, including *BRD4* harbored putative binding sites complementary to the seed sequence of miR-211-5p (Supplementary Table 1). It was found that the miR-211-5p binding sites on SNHG18 and *BRD4* 3’-UTR were overlapped (Fig. 7A). Luciferase reporter assay demonstrated that overexpression of miR-211-5p significantly suppressed the expression of the *BRD4* 3’-UTR reporter (Fig. 7B). The endogenous levels of BRD4 transcript in A549 and H1299 cells were consistently reduced after ectopic expression of miR-211-5p (Fig. 7C). These observations indicate that BRD4 is a novel target of miR-211-5p.

Several previous studies have revealed the oncogenic role of BRD4 in NSCLC^28,29^. We then asked whether downregulation of BRD4 is required for the activity of miR-211-5p. We found that enforced expression of BRD4 (lacking the 3’-UTR) rescued the inhibitory effects of miR-211-5p on NSCLC cell proliferation and invasion (Fig. 7D–F).

### Derepression of BRD4 contributes to SNHG18-mediated aggressive phenotype

**Fig. 8:**
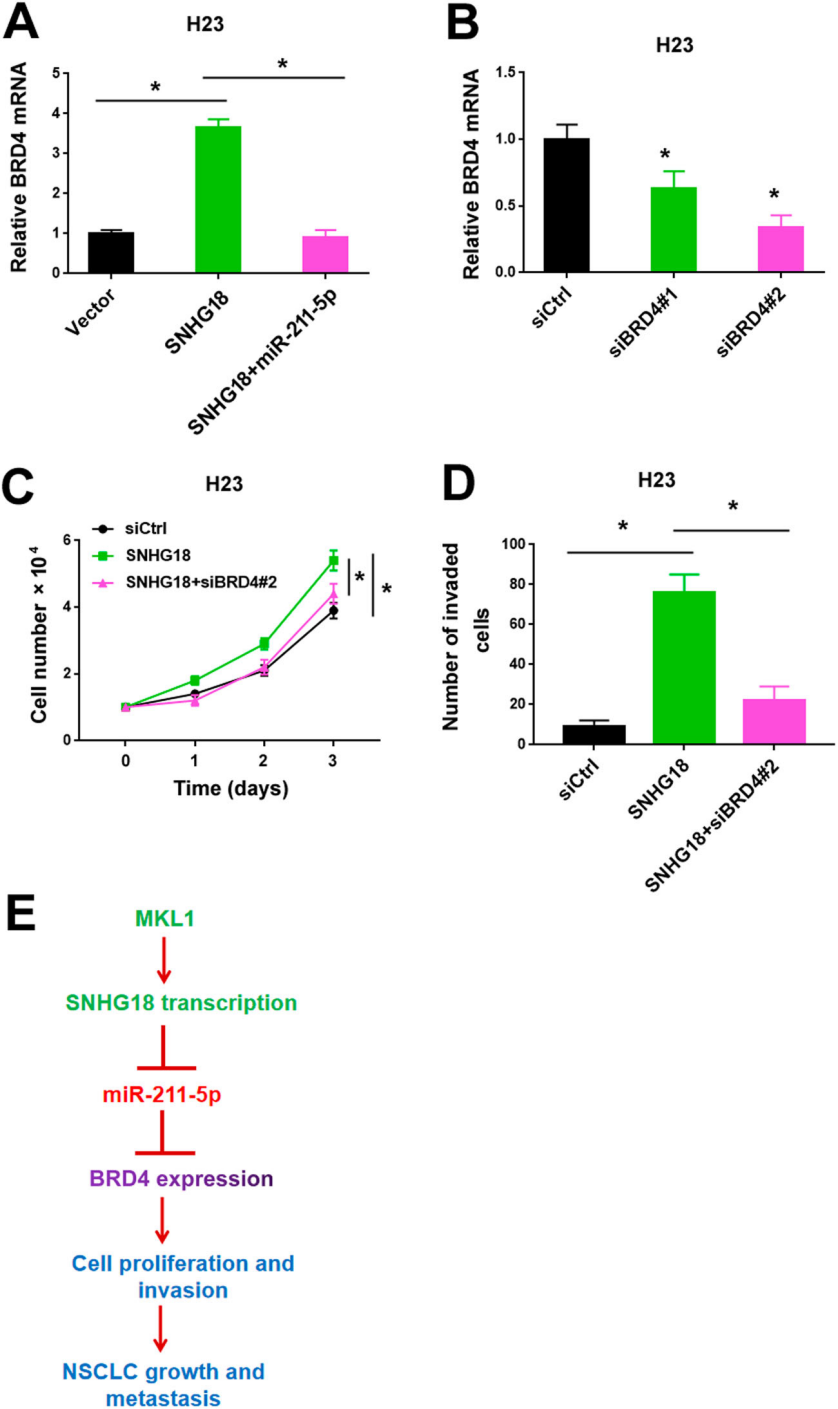
Derepression of BRD4 contributes to SNHG18-mediated aggressive phenotype. **A** Quantitative PCR analysis of BRD4 mRNA in H23 cells transfected with indicated constructs. *n* = 3. **B** Quantitative PCR analysis of BRD4 mRNA in H23 cells transfected with control siRNA (siCtrl) or 2 different BRD4-targeting siRNAs (siBRD4#1 and siBRD4#2). *n* = 3. **C**, **D** Assessment of **C** cell proliferation (*n* = 4) and **D** invasion (*n* = 3) in H23 cells transfected with indicated constructs. In all panels in this figure, one-way ANOVA and Bonferroni’s tests were performed. *P < 0.05. **E** Schematic diagram of the molecular mechanism by which MKL1-induced SNHG18 promotes NSCLC growth and metastasis through the miR-211-5p/BRD4 pathway.

Our data showed that overexpression of SNHG18 resulted in an upregulation of BRD4 in H23 cells (Fig. 8A). Co-expression of miR-211-5p reversed the induction of BRD4 by SNHG18. Next, we explored whether SNHG18-mediated aggressiveness is associated with the derepression of BRD4. We found that silencing of BRD4 (Fig. 8B) blocked SNHG18-induced proliferation (Fig. 8C) and invasion (Fig. 8D) in H23 cells.

## Discussion

Our results indicate SNHG18 as an MKL1-induced lncRNA in NSCLC cells. SNHG18 plays an essential role in mediating MKL1-induced NSCLC cell growth and invasion. Depletion of SNHG18 blocks NSCLC tumorigenesis and metastasis in vivo. The tumor-promoting activity of SNHG18 is largely attributed to antagonization of miR-211-5p and upregulation of BRD4. Silencing of BRD4 reverses the effects of SNHG18 on NSCLC cell proliferation and invasion. These observations collectively indicate that SNHG18 contributes to MKL1-induced aggressiveness in NSCLC cells through modulation of the miR-211-5p/BRD4 pathway.

MKL1 is capable of upregulating many genes involved in cancer development and progression^7,23,30^. In breast cancer, MKL1 together with SRF promotes the expression of IL-11 to enhance breast cancer stemness and metastasis^30^. MKL1 recruits Smad3 to transactivate MMP2 in papillary thyroid cancer, consequently accelerating nodal metastasis^7^. Similarly, MKL1 reinforces NSCLC aggressiveness by increasing the transcription of MMP9^23^. Several non-coding genes such as miR-206 and HOTAIR can also be transactivated by MKL1^31,32^. However, the downstream target genes mediating the tumor-promoting activity of MKL1 in NSCLC are not clearly defined. In this study, we identify a novel MKL1-regulated non-coding gene and show that SNHG18 expression in NSCLC cells is induced by MKL1. ChIP assay demonstrates the occupancy of MKL1 protein on the promoter of *SNHG18*. Luciferase reporter assay confirm that the region (−1400 to −900 bp) of *SNHG18* promoter is essential for MKL1-mediated transactivation of SNHG18 (Fig. 1). These results suggest SNHG18 as a downstream target gene of MKL1. However, MKL1 alone appears not to be sufficient to initiate the transcription of SNHG18. When the *SNHG18* promoter-luciferase construct is transfected together with MKL1-expressing plasmid to 293T cells, no significant luciferase activity was produced (data not shown). Therefore, MKL1 plays an essential but not sufficient role in the transactivation of SNHG18.

A number of SNHGs have shown the ability to regulate lung cancer progression^33–35^. For instance, SNHG1 promotes NSCLC migration and invasion by sponging miR-145-5p^33^. SNHG3 potentiates the proliferation and migration of NSCLC cells through the TGF-β and IL-6/JAK2/STAT3 pathways^34^. SNHG15 accelerates NSCLC metastasis by upregulating CDK14 via inhibition of miR-486^35^. In this study, we provide the first evidence for the oncogenic role of SNHG18 in NSCLC. Our data show that knockdown of SNHG18 blocks the proliferation, colony formation, and invasion in vitro and tumorigenesis and metastasis of NSCLC cells in vivo (Figs. 3 and 4). Conversely, ectopic expression of SNHG18 reinforces the proliferation and invasion of NSCLC cells (Fig. 5). In support of the oncogenic role of SNHG18 in NSCLC cells, upregulation of SNHG18 is associated with advanced disease and poor patient prognosis (Fig. 2). Most importantly, depletion of SNHG18 impairs MKL1-induced NSCLC cell proliferation and invasion (Fig. 3F, G). Taken together, SNHG18 is involved in MKL1-mediated aggressive phenotype in NSCLC cells.

Mechanistic investigation reveals that SNHG18 suppresses miR-211-5p to upregulate BRD4 in NSCLC cells. Interaction with miRNAs is an important mechanism by which lncRNA exerts its biological activity^13,14^. It is notable that SNHG18 selectively inhibits miR-211-5p in NSCLC cells. Consistently, an inverse correlation between SNHG18 and miR-211-5p is observed in NSCLC tissues. Furthermore, both SNHG18 and miR-211-5p are loaded in the Ago2 complex isolated from NSCLC cells. These data suggest that SNHG18 interacts with miR-211-5p (Fig. 6A–C). Functionally, abrogation of miR-211-5p recapitulates SNHG18 overexpression phenotype in cell proliferation and invasion. Moreover, ectopic expression of miR-211-5p attenuates SNHG18-induced NSCLC cell proliferation and invasion. Previously, miR-211-5p has been reported to act as a tumor suppressor in renal cancer, breast cancer, and tongue cancer^36–38^. Here, we provide evidence that downregulation of miR-211-5p is involved in the tumor-promoting activity of SNHG18 in NSCLC (Fig. 6D–G).

The activity of miR-211-5p in different cancer types is mediated through the downregulation of specific target genes. For instance, miRNA-211-5p inhibits tumor cell proliferation and invasion in breast cancer by targeting SETBP1^37^. Similarly, miRNA-211-5p exerts its anti-invasive activity in renal cancer by targeting SNAI1^36^. However, we did not observe any effect on the expression of SETBP1 or SNAI1 in NSCLC cells overexpressing miR-211-5p (data not shown). Instead, we find that overexpression of miR-211-5p suppresses BRD4 expression in NSCLC cells (Fig. 7A–D). Previously, it has been documented that BRD4 is capable of promoting cancer cell growth and invasion^28,29,39^. Here, we show that SNHG18 can enhance the expression of BRD4, and miR-211-5p prevents SNHG18-mediated upregulation of BRD4. Enforced expression of BRD4 rescues miR-211-5p phenotype on NSCLC cell proliferation and invasion, while silencing of BRD4 impairs SNHG18-induced aggressiveness in NSCLC cells (Fig. 7E and F). Altogether, these findings suggest that SNHG18 regulates the miR-211-5p/BRD4 axis to enhance NSCLC growth and metastasis (Fig. 8E).

Although in this study, we show that SNHG18 is required for MKL1-dependent aggressive phenotype of NSCLC cells, other lncRNAs might also contribute to the tumor-promoting activity of MKL1. Future experiments based on transcriptomic profiling of lncRNAs will provide more insight into the mechanism by which MKL1 induces lncRNAs to enhance NSCLC growth and invasion.

In conclusion, our study demonstrates that SNHG18 is an MKL1-regulated lncRNA and plays an oncogenic role in NSCLC. SNHG18 promotes NSCLC growth and metastasis through the miR-211-5p/BRD4 axis. Therefore, targeting SNHG18 may provide a novel therapeutic strategy against NSCLC.

## Supplementary information

Supplementary data
