# Supplementary material for: MKL1-induced lncRNA SNHG18 drives the growth and metastasis of non-small cell lung cancer via the miR-211-5p/BRD4 axis
Source: Cell Death Dis. 2021 Jan 26;12(1):128. doi: 10.1038/s41419-021-03399-z (PMC7838315; doi:10.1038/s41419-021-03399-z)
Supplement: Supplementary file 1 — Supplementary data [file 41419_2021_3399_MOESM1_ESM.doc]

**Supplementary Table S1. Sequences of PCR primers used in this study.**

| **Name** | **Direction** | **Primer sequences (5′ to 3′)** | **Assay** |
| --- | --- | --- | --- |
| SNHG18 | Forward | CCTAATGCTAAACATTGGTACA | Quantitative PCR |
|  | Reverse | GCAACACAGCATCACCTGTAC |  |
| TINCR | Forward | GAGTCCTGTTGGCTGCAGGAC | Quantitative PCR |
|  | Reverse | AGTTTGGCAGATGCTCTGAAGG |  |
| TTN-AS1 | Forward | GGGAGTCAGCTTTACTGGTG | Quantitative PCR |
|  | Reverse | TCCCGATGCTGAGTATCAGTTC |  |
| MKL1 | Forward | CCAAGTCTGCCAGTGAGAAGTC | Quantitative PCR |
|  | Reverse | GCTGGAGGAAGAGCTGCTGCTG |  |
| BRD4 | Forward | CCAAATGTCTACACAGTATACA | Quantitative PCR |
|  | Reverse | GGTCTGTTCAAGGCAAAGTCA |  |
| GAPDH | Forward | ACCACAGTCCATGCCATCAC | Quantitative PCR |
|  | Reverse | ACCACCCTGTTGCTGTA |  |
| U6 | Forward | CTCGCTTCGGCAGCACA | Quantitative PCR |
|  | Reverse | AACGCTTCACGAATTTGCGT |  |
| MKL1 | Forward | GGGGATCCATGCCGCCTTTGAAAAGTCCA | Cloning |
|  | Reverse | GGGAATTCCTACAAGCAGGAATCCCAGTG |  |
| SNHG18 | Forward | GGGGATCCGCCGGGGTGAAACTCGAGC | Cloning |
|  | Reverse | GGGAATTCCAGATTTCTAGAATCCTTTAATTG |  |
| BRD4 | Forward | GGGGATCCATGTCTGCGGAGAGCGGCCCT | Cloning |
|  | Reverse | GGCTCGAGTCAGAAAAGATTTTCTTCAAATATTG |  |
| *SNHG18* promoter | Forward | CGGTGTGCAAAATCCGTGGA | ChIP |
|  | Reverse | GGCAGGTTTTCCTGCTTTCCT |  |
| *GAPDH* promoter | Forward | CGGGATTGTCTGCCCTAATTAT | ChIP |
|  | Reverse | GCACGGAAGGTCACGATGT |  |

**Supplementary Table S2. Targeting sequences for siRNAs and shRNAs and miRNA oligonucleotides.**

| **Gene** | **ID** | **Targeting sequences (5′ to 3′)** |
| --- | --- | --- |
| MKL1 | shRNA#1 | TGCACATTTTGGAAGAGACCT |
|  | shRNA#2 | GAGCCTATCAAGACCAAATCA |
| SNHG18 | shRNA#1 | CTTTCTGGAAAGAACATCACC |
|  | shRNA#2 | ACTGAAGAAATGGAAAGATCA |
| BRD4 | siRNA#1 | UGAGUACCGUGAUGCUCA |
|  | siRNA#2 | CUGAUUACUAUAAGAUCAU |

**Supplementary Table S3. miR-211-5p mimics and anti-miR-211-5p inhibitor.**

| **Name** | **Catalog #** | **Company** |
| --- | --- | --- |
| miR-211-5p mimic | HMI0374 | Sigma-Aldrich |
| Anti-miR-211-5p | 4464084 | Thermo Fisher Scientific |


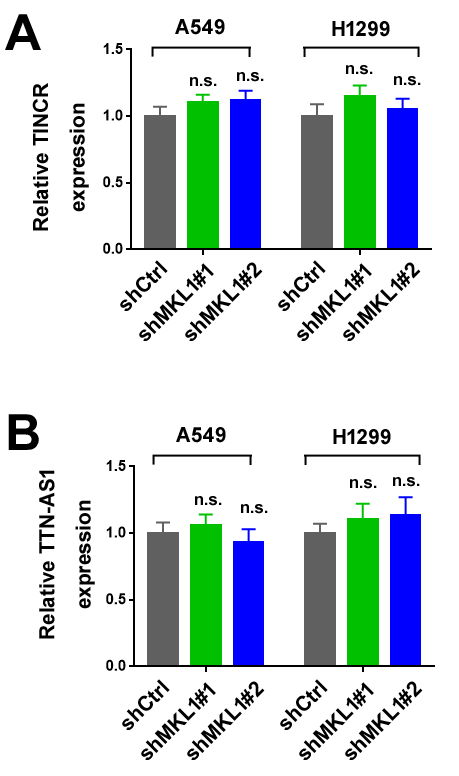


Supplementary Figure S1. Effect of MKL1 knockdown on the expression of TINCR and TTN-AS1 in A549 and H1299 cells. Quantitative PCR analysis of (A) TINCR and (B) TTN-AS1 expression in cells transfected with negative control shRNA (shCtrl) or 2 different shRNAs targeting MKL1 (shMKL1#1 and shMKL1#2). n.s. indicates no significance.
